# Attofarad-Class Ultra-High-Capacitance Resolution Capacitive Readout Circuits

**DOI:** 10.3390/s25082461

**Published:** 2025-04-14

**Authors:** Guoteng Ren, Saifei Yuan, Jingjing Peng, Ruitao Liu, Yuhao Feng, Haonan Liu, Wenshuai Lu, Fei Xing, Ting Sun, Shijie Yu

**Affiliations:** 1 College of Instrument Science and Opto-Electronics Engineering, Beijing Information Science and Technology University, Beijing 100192, China; 2023020156@bistu.edu.cn (G.R.); feng_yuhao22@bistu.edu.cn (Y.F.); 2023020357@bistu.edu.cn (H.L.); sunting@bistu.edu.cn (T.S.); 2 Laboratory of Intelligent Microsystems, Beijing Information Science and Technology University, Beijing 100192, China; 3 State Key Laboratory of Precision Measurement Technology and Instruments, Tsinghua University, Beijing 100095, China; yuasaifei@163.com (S.Y.); pjj.csu.mat@163.com (J.P.); yuansaifei2022@163.com (R.L.); luwenshuai_dky@163.com (W.L.); 4 Department of Precision Instrument, Tsinghua University, Beijing 100084, China; xingfei@mail.tsinghua.edu.cn

**Keywords:** accelerometer, high-resolution capacitance, low noise, readout circuit

## Abstract

In order to meet the application requirements for high-precision and low-noise accelerometers in micro-vibration measurement and navigation fields, this paper presents the design and testing of an ultra-high-capacitance resolution capacitive readout circuit with attofarad-level precision. First, a differential charge amplifier circuit is employed for the first stage of capacitance detection. To suppress noise interference in the circuit, a frequency-domain modulation technique is utilized to mitigate low-frequency noise. Subsequently, a differential subtraction circuit is implemented to reduce common-mode noise. Additionally, an improved filtering circuit is designed to suppress noise interference in the final stage. The test results indicate that the designed circuit operates at a carrier frequency of 1 MHz, achieving a capacitance resolution of up to 0.103 aF/H$z^{1/2}$ and a noise floor of 25.6 μg/H$z^{1/2}$, thereby meeting the requirements for high-precision and low-noise capacitance detection in MEMS accelerometers.

## 1. Introduction

MEMS capacitive accelerometers, which offer advantages such as compact size and low power consumption, are widely utilized in various applications, including unmanned system motion control, platform stabilization, and navigation [1,2]. To expand their application range, especially for miniature wearable devices, further miniaturization of the devices is required. Due to the third-order miniaturization effect, the variation in equivalent capacitance decreases to the attofarad (aF) level, making detection and control more challenging. Furthermore, in fields like micro-vibration measurement, there is an increasing demand for capacitance detection signals to have a high signal-to-noise ratio (SNR). Detecting weak signals is critical for achieving high-SNR capacitance detection. As a result, the design of capacitive readout circuits with high-capacitance resolution has become a significant research focus in the field of MEMS accelerometer detection.

Currently, the main implementation methods for capacitance-to-voltage (C-V) conversion circuits include continuous-time voltage (CTV) readout circuits [3], switched-capacitor (SC) detection circuits [4,5,6,7,8], and continuous-time current (CTC) readout circuits [9]. In the CTV structure, it is crucial to provide a stable and highly reliable DC biasing circuit. To avoid signal attenuation, the resistance of the biasing circuit must be very high. However, large resistors not only occupy significant chip area but also generate considerable parasitic capacitance. In this structure, the large parasitic capacitance can lead to a reduction in circuit sensitivity. The SC circuit structure provides virtual ground for the output node of the micro-accelerometer through periodic reset switches [10,11,12]. The detection signal is insensitive to parasitic capacitance $C_{P}$ and charge accumulation effects. However, due to the limitations of the switches themselves, clock feedthrough and charge injection from the switches further degrade noise performance. The noise charge is stored in the parasitic capacitance $C_{P}$, and the resulting gain degradation exacerbates signal distortion. To compensate for these issues, the scale and power consumption of SC circuits are increased further [13]. In contrast, in the CTC structure, the charge amplifier offers advantages such as low circuit noise and high sensitivity, making it suitable for high-precision detection scenarios. However, parasitic capacitance at the input of the charge amplifier, common-mode ground offset, resistor-capacitor device errors, and other factors impact detection accuracy, serving as limiting factors for the detection capability of charge amplifiers.

To improve detection accuracy, this paper presents the design of a capacitive readout circuit. The circuit utilizes a differential charge amplifier to achieve capacitance-to-voltage conversion. Frequency-domain modulation techniques are employed to suppress low-frequency noise interference, while a high-precision common-mode bias is used as a virtual ground to shield against disturbances caused by poor grounding. The final-stage filter adopts an infinite gain structure, which effectively suppresses the voltage accuracy degradation caused by resistor-capacitor errors in the final-stage filter. The circuit is simulated using LTspice (v. 24.0.12), followed by PCB verification. The scale factor, zero-bias stability, and noise performance are measured experimentally. The test results show that the proposed design excels at suppressing low-frequency noise and common-mode interference, achieving high-precision detection of weak capacitance variations in the accelerometer.

The rest of this paper is organized as follows. Section 2 presents the structure and measurement principles of the accelerometer. Section 3 details the design scheme and working principles of the capacitive readout circuit. Section 4 analyzes the circuit noise and derives an optimized design. Section 5 presents the measurements and comparisons with other designs. Finally, Section 6 concludes this article.

## 2. Accelerometer Structure and Measuring Principle

**Figure 1:**
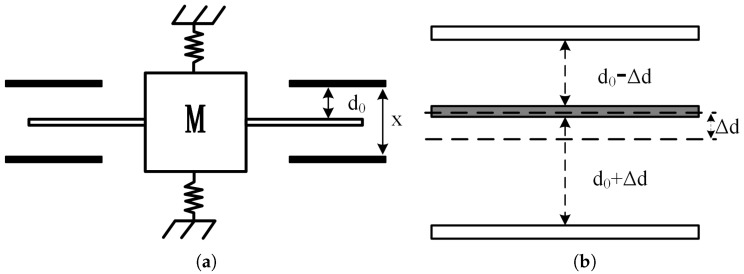
Schematic diagram of accelerometer principle: (**a**) MEMS accelerometer model. (**b**) Movable pole plate displacement.

The MEMS capacitive accelerometer consists of fixed parallel plates, a movable proof mass, and beams, as illustrated in Figure 1a. The space between the plates can be modeled as an equivalent capacitance. In the absence of an acceleration signal, the distance between the central plate and the upper and lower stator plates is $d_{0}$, resulting in an equivalent capacitance of $C_{0}$. When an acceleration signal is applied, inertial forces cause displacement of the plates, as shown in Figure 1b. Let the displacement of the movable plate be *x*. Consequently, the effective distance between the upper stator plate and the central plate becomes $d_{0}-x$, while the effective distance between the lower stator plate and the central plate becomes $d_{0}+x$. Based on these displacement conditions, the resulting capacitance change, ΔC, is given by

(1) $$\Delta C=C_{1}-C_{2}=\left(\frac{1}{1-\frac{x}{d_{0}}}-\frac{1}{1+\frac{x}{d_{0}}}\right)\cdot C_{0}$$

Based on the relationship between the capacitance change ΔC and the displacement x of the plates, using Taylor’s formula, the relationship between the output capacitance variation in the accelerometer and the plate displacement can be approximated by

(2) $$\Delta C=2C_{0}\left[\frac{x}{d}+{\left(\frac{x}{d}\right)}^{3}+{\left(\frac{x}{d}\right)}^{5}+\cdots +{\left(\frac{x}{d}\right)}^{n}\right]\approx 2C_{0}\frac{x}{d_{0}}$$

When the accelerometer is in a steady state under an applied force, the proof mass remains stable, and the displacement x of the comb fingers becomes a constant. According to the mechanical model of the MEMS accelerometer, all higher-order derivatives of x with respect to time are zero. Therefore, according to Hooke’s law and Newton’s second law of motion, we have kx=ma. Combining with Equation (2), we obtain

(3) $$a\approx \Delta C\frac{d_{0}k}{2mC_{0}}$$

Therefore, when x≪d, the external acceleration is approximately linearly related to the variation in the differential capacitance. Hence, the actual acceleration value can be obtained by detecting the change in the differential capacitance of the accelerometer. This capacitance variation requires a high-precision capacitive readout circuit for detection, where the circuit output voltage is linearly related to the acceleration, thereby enabling accurate acceleration measurement.

## 3. Capacitive Readout Circuit Design

**Figure 2:**
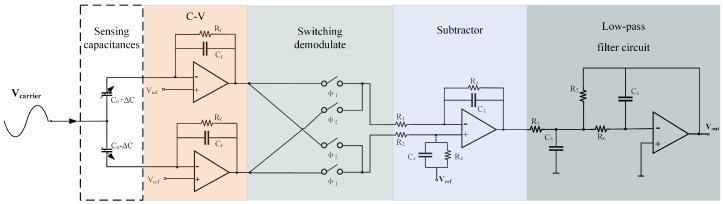
The overall structure of the capacitive readout circuit.

A differential charge amplifier is used to implement C-V conversion in this paper. This circuit structure allows the measurement of small capacitance variations, even in the presence of large parasitic capacitance to ground. Additionally, the differential structure ensures that the non-inverting input voltage remains stable, and signal modulation can be achieved with a single carrier. The capacitive readout circuit is shown in Figure 2. The high-frequency carrier signal $V_{carrier}$ modulates the capacitance signal to a high frequency through the accelerometer. The C-V conversion circuit then converts the differential capacitance changes into voltage. After demodulation by the switch, the signal of interest is downconverted to low frequency, while the noise remains at high frequency. After subtraction, common-mode noise in the signal is further eliminated. Finally, after passing through a low-pass filter, the high-frequency noise is removed, yielding the high-precision acceleration signal to measure.

**Figure 3:**
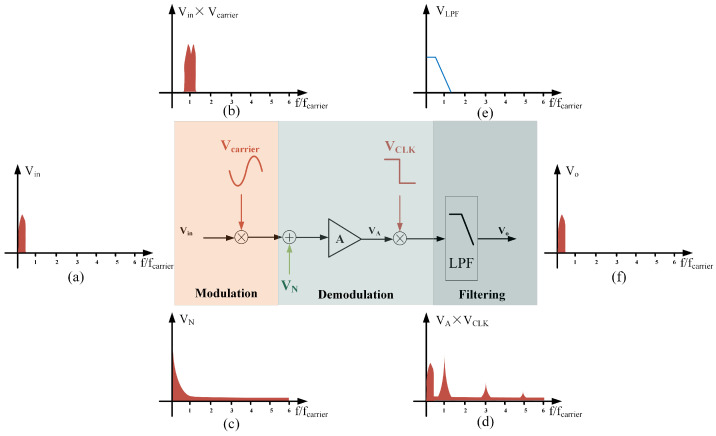
Signal spectrum graph. (**a**) Spectrum of the signal of interest. (**b**) Spectrum of the modulated signal of interest. (**c**) Noise spectrum in the circuit. (**d**) Demodulated spectrum of the signal of interest and noise. (**e**) Spectrum of the low-pass filter. (**f**) Spectrum of the output signal.

To more intuitively illustrate the circuit’s suppression effect on low-frequency noise, the circuit structure is simplified, as shown in Figure 3. In the modulation stage, the signal of interest is modulated to the carrier frequency, as shown in (b), while the noise in the circuit remains in the low-frequency band, as shown in (c). After the C-V conversion module, the signal is demodulated, transferring the signal of interest to a low frequency, while the low-frequency noise is modulated to a high frequency, as shown in (d). After passing through the low-pass filter, as shown in (e), the final output is the high-precision signal of interest, as depicted in (f).

### 3.1. Capacitor-Voltage Conversion Circuit

**Figure 4:**
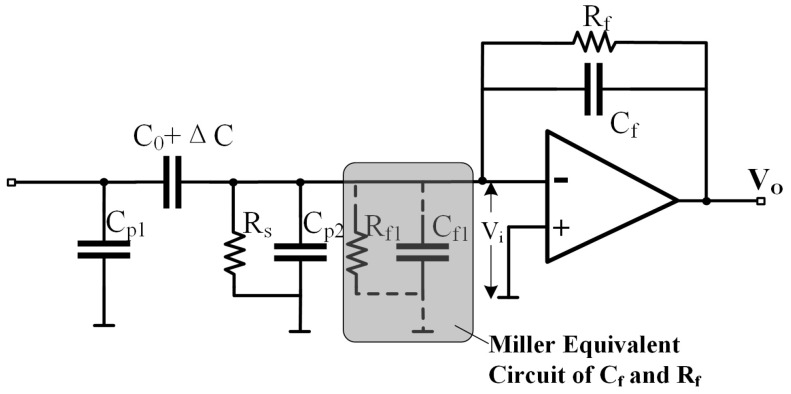
Charge amplifier equivalent circuit.

The charge amplifier can perform the first stage of capacitance detection. First, the equivalent circuit of a typical charge amplifier is analyzed, as shown in Figure 4. In the figure, $C_{0}$ represents the equivalent static capacitance, ΔC is the capacitance variation, $R_{s}$ is the input resistance of the operational amplifier, $V_{carrier}$ is the carrier signal, $C_{p1}$ and $C_{p2}$ are the parasitic capacitance values at the interface, $R_{f}$ is the feedback resistor, $C_{f}$ is the feedback capacitor, and $R_{f1}$ and $C_{f1}$ are the Miller equivalent values of the feedback resistor and feedback capacitor, respectively.

As argued in [14,15], the impact of $C_{p1}$ on the circuit is minimal because it is charged and discharged by a low-impedance source. Therefore, its effect can be neglected when calculating the output voltage $V_{o}$. The final relationship between $V_{o}$ and ΔC is as follows:

(4) $$\begin{array}{cc} V_{i} & =-V_{carrier}\left(\frac{R_{f}}{1+A}//\left(1+A\right)C_{f}//R_{s}//C_{p2}\right).\\ \; & {\left(\frac{1}{j\omega (C_{o}+\Delta C)}+\frac{R_{f}}{1+A}//\left(1+A\right)C_{f}//R_{s}//C_{p2}\right)}^{-1} \end{array}$$

(5) $$V_{o}=-AV_{i}\approx -V_{carrier}\frac{C_{0}+\Delta C}{C_{f}}$$

When the operational amplifier’s open-loop gain *A* and its equivalent input resistance are sufficiently large and the conditions $\left(1+A\right)\left(1/R_{f}+j\omega C_{f}\right)\gg \left(1/R_{s}+j\omega C_{P}\right)$ and $R_{f}\gg 1/\omega C_{f}$ are satisfied, the output voltage exhibits a linear relationship with ΔC.

**Figure 5:**
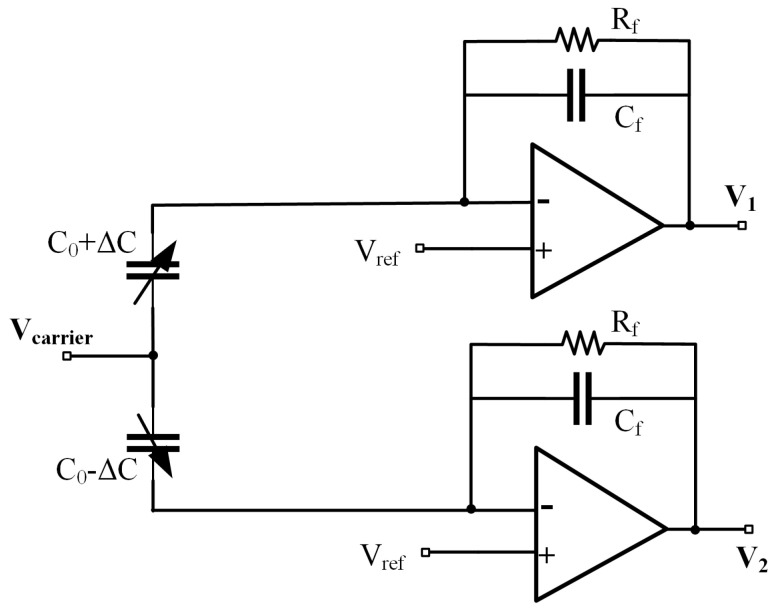
Differential charge amplifier structure.

This design uses a differential charge amplifier to implement the first stage of capacitance detection, thereby improving detection accuracy and achieving dual outputs. After passing through a subtraction circuit, common-mode noise interference is effectively suppressed. The circuit structure is shown in Figure 5. The input high-frequency carrier signal is $V_{carrier}=V_{P}+V_{ref}$, and the sine wave signal is labeled $V_{P}$. $V_{ref}$ represents the reference voltage at the inverting input of the operational amplifier, ensuring that the charge amplifier operates in a deep negative feedback state to maintain the circuit’s stability and linear response.

Based on the typical output equation of the charge amplifier circuit shown in Equation (5), when the condition $R_{f}\gg 1/\omega C_{f}$ is met, the output voltages $V_{1}$ and $V_{2}$ are linearly related to the capacitors $C_{1}$ and $C_{2}$, ensuring accurate detection, as follows:

(6) $$\left\{\begin{array}{c} V_{1}=V_{ref1}-V_{P}\cdot \frac{C_{1}}{C_{f}}\\ V_{2}=V_{ref1}-V_{P}\cdot \frac{C_{2}}{C_{f}} \end{array}\right.$$

### 3.2. Demodulation Circuit

**Figure 6:**
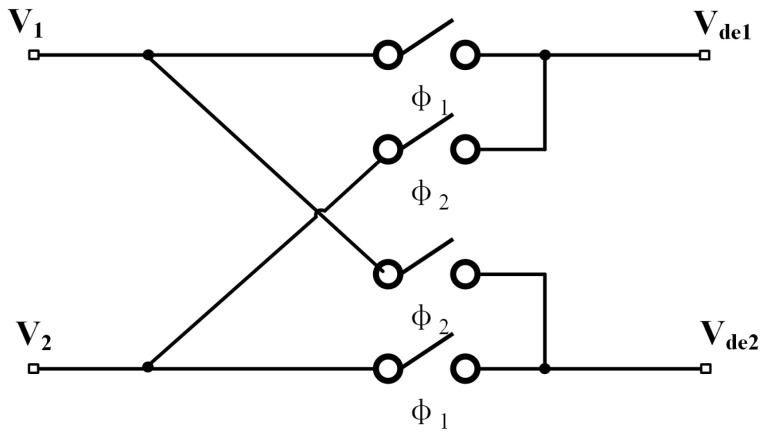
Structure of the demodulation circuit.

By introducing a demodulation signal that is in phase and frequency with the modulation signal $V_{carrier}$, the signal of interest is demodulated to the low-frequency band, thereby suppressing interference from the circuit’s low-frequency noise. The demodulation circuit depicted in Figure 6 utilizes an analog switch to perform the demodulation function. The clock signal that controls the switching of the analog switch is a square-wave signal that is in phase and frequency with the input carrier signal, which allows for signal selection. This is equivalent to multiplying the modulation signal by a square-wave reference signal with an amplitude of ±1.

The square-wave signal is expanded using a Fourier series, as shown in the following equation:

(7) $$V_{CLK}\left(t\right)=\frac{4}{\pi }\sum_{n=1}^{\infty }\frac{1}{2n-1}\sin\left[\left(2n-1\right)\omega_{0}t\right]$$

The input modulating signal is $V_{P}\left(t\right)=Asin\left(\omega_{0}t\right)$, where *A* is the sinusoidal amplitude and $\omega_{0}$ is the angular frequency. The switching demodulated output is

(8) $$\begin{array}{cc} V_{f} & =A\sin\left(\;\omega_{0}t\right)\times \frac{4}{\pi }\sum_{n=1}^{\infty }\frac{1}{2n-1}\sin\left[\left(2n-1\right)\omega_{0}t\right]\\ \; & =\frac{2}{\pi }A\sum_{n=1}^{\infty }\frac{1}{2n-1}\cos\left[\left(2n-2\right)\omega_{0}t\right]-\\ \; & \frac{2}{\pi }A\sum^{\infty }\frac{1}{2n-1}cos\left[2n\omega_{0}t\right] \end{array}$$

The demodulated output is passed through the low-pass filter circuit, and the high-frequency components are filtered out, leaving only the difference-frequency term for n = 1, as shown in Equation (9). Ignoring the high-frequency components, the output voltage of the demodulation circuit is given in Equation (10):

(9) $$V_{f}\left(t\right)=\frac{2}{\pi }A$$

(10) $$\left\{\begin{array}{c} V_{de1}=V_{ref1}\cdot V_{CLK}\left(t\right)-\frac{2}{\pi }A\cdot \frac{C_{1}}{C_{f}}\\ V_{de2}=V_{ref2}\cdot V_{CLK}\left(t\right)-\frac{2}{\pi }A\cdot \frac{C_{2}}{C_{f}} \end{array}\right.$$

### 3.3. Subtraction Circuit

**Figure 7:**
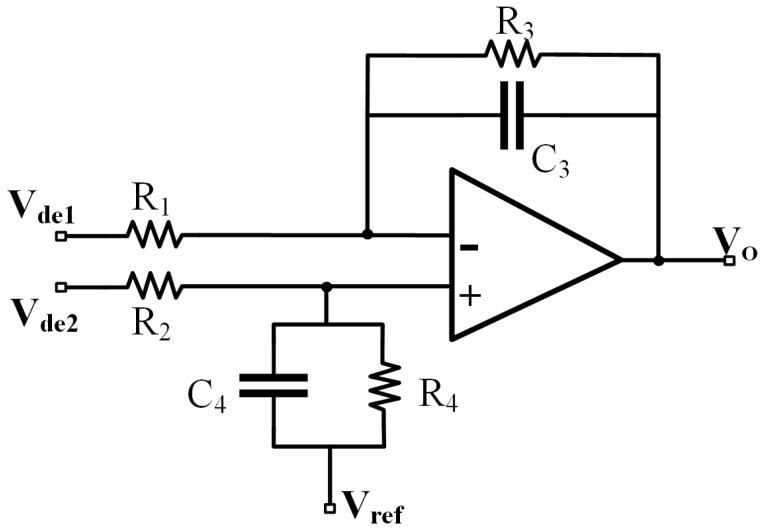
Structure of subtraction circuit.

The common-mode noise interference in the two outputs of the demodulation circuit is eliminated through a subtraction circuit while obtaining the output signal that is related to ΔC. The subtraction circuit is composed of a differential amplifier, and the circuit is shown in Figure 7.

The output voltage signal $V_{o}$ of the subtraction circuit is

(11) $$V_{o}=\left(V_{de2}-V_{de1}\right)\frac{R_{3}}{R_{1}\left(1+j\omega R_{3}C_{3}\right)}+V_{ref}$$

It can be seen that through the subtraction circuit, the differential-mode signal is amplified, and the common-mode signal is suppressed, effectively reducing the common-mode interference signal. At the same time, the subtraction circuit also has the effect of low-pass filtering. You can adjust the amplification through $R_{1}$ and adjust the filtering effect through $R_{3}$ and $C_{3}$. The calculation of the output signal of the subtraction circuit $V_{o}$ is

(12) $$V_{o}=\frac{2A}{\pi }\cdot \frac{\Delta C}{C_{f}}\cdot \frac{R_{3}}{R_{1}}+V_{ref}$$

### 3.4. Filter Circuit

**Figure 8:**
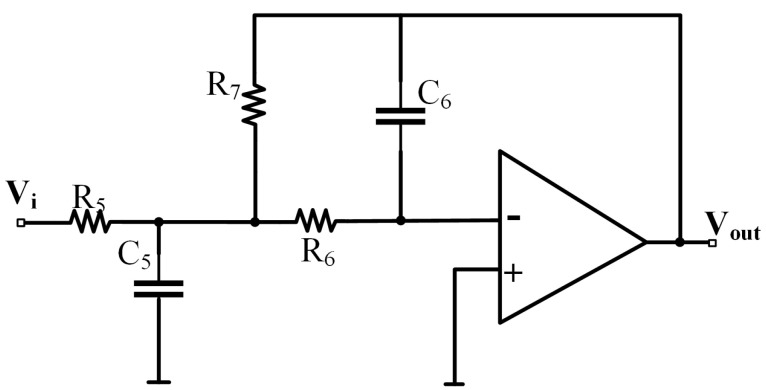
Structure of MFB low-pass filtering circuit.

The signal from the demodulation circuit includes low-frequency signals to be measured and high-frequency noise signals. To obtain high-precision low-frequency signals, the high-frequency noise signals in the circuit need to be filtered out. To avoid a shift in the cutoff frequency due to the resistance-capacitance error of the end-stage stage filter, which leads to a decrease in the accuracy of the output signal, the low-pass filter uses a high Q-value and low resistance-capacitance error sensitivity, achieved through the infinite-gain multi-feedback (MFB) filter, as shown in Figure 8.

The filter transfer function can be obtained as follows:

(13) $$\frac{V_{out}}{V_{in}}=-\frac{R_{7}/R_{5}}{R_{6}R_{7}C_{5}C_{6}s^{2}+\left(R_{6}+R_{7}+\frac{R_{6}R_{7}}{R_{5}}\right)C_{6}s+1}$$

According to the filter transfer function, the filter amplification is $R_{7}/R_{5}$. To avoid amplifying the noise signal, assume $R_{7}=R_{5}$ so that the filter amplification is 1. The final circuit output voltage $V_{out}$, which is the output of the subtraction circuit $V_{o}$, as shown in Equation (13), illustrates that the output of the capacitive readout circuit $V_{out}$ varies linearly with the amount of change ΔC.

## 4. Circuit Noise Analysis and Optimized Design

**Figure 9:**
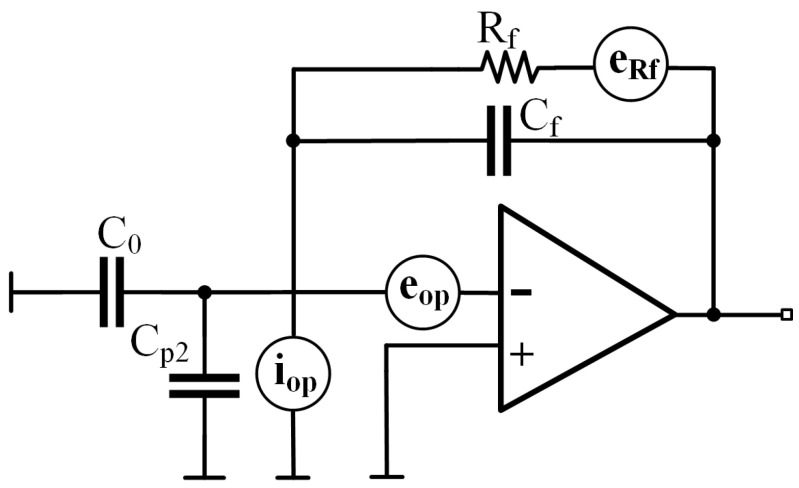
Equivalent noise model of capacitive readout circuit.

The noise performance of the circuit mainly depends on the noise of the first-stage amplifier. Reducing the noise of the first-stage amplifier and optimizing the noise transfer function can effectively lower the system noise [16,17]. For C-V circuits employing differential charge amplifiers with symmetrical configurations, it is sufficient to analyze a single circuit. To simplify the analysis, the small resistor and capacitor values of $R_{s}$, $C_{p1}$, and ΔC are not taken into account. Its equivalent noise model is shown in Figure 9 and mainly includes the operational amplifier’s input-referred noise voltage $e_{op}$, the operational amplifier’s input-referred noise current $i_{op}$, and the resistor thermal noise $e_{Rf}$. The noise density expression of the resistor thermal noise is given by $\sqrt{4kTR}$, where *k* is the Boltzmann constant, *T* is the absolute temperature, and *R* is the resistance value.

Assuming that the noise sources are not related to each other and using the principle of linear superposition to calculate the contribution of each noise source to the output noise, the output noise power spectral density can be obtained as follows:

(14) $$\begin{array}{cc} e_{1}^{2} & =e_{Rf}^{2}\cdot |H_{1}{\left(j\omega \right)|}^{2}+e_{op}^{2}\cdot |H_{2}{\left(j\omega \right)|}^{2}+i_{op}^{2}\cdot {\left|H_{3}\left(j\omega \right)\right|}^{2}\\ & =e_{Rf}^{2}{\left(\frac{1}{1+j\omega R_{f}C_{f}}\right)}^{2}+e_{op}^{2}{\left(\frac{1+j\omega R_{f}C_{i}}{1+j\omega R_{f}C_{f}}\right)}^{2}+i_{op}^{2}{\left(\frac{R_{f}}{1+j\omega R_{f}C_{f}}\right)}^{2} \end{array}$$

where $H_{1}$, $H_{2}$, and $H_{3}$ correspond to the transfer functions from $e_{Rf}$, $e_{op}$, and $i_{op}$ to the output, respectively, with $C_{i}=C_{0}+C_{f}+C_{p2}$.

After using the modulation-demodulation method to suppress low-frequency 1/f noise, the high-frequency part of the readout circuit noise becomes the main factor limiting its resolution. In Equation (14), it can be seen that resistor thermal noise and op-amp current noise both decrease with increasing circuit operating frequency. When the operating frequency exceeds a certain threshold, the contribution of resistor thermal noise and op-amp current noise to the output noise of the circuit is approximately negligible compared to the op-amp voltage noise. At this point, the above equation can be simplified to

(15) $$e_{1}^{2}={\left|\frac{C_{0}+C_{p2}}{C_{f}}+1\right|}^{2}e_{op}^{2}$$

**Figure 10:**
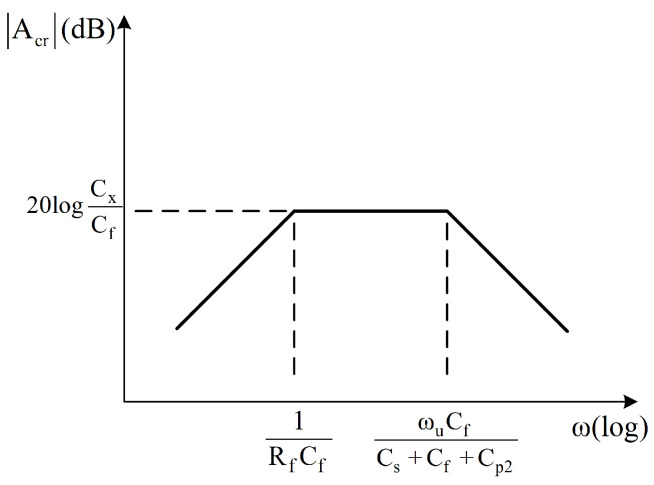
The Bode plot of the C-V conversion circuit.

Under high-frequency conditions, the input noise voltage source of the op-amp gradually becomes the main source of noise, and $C_{p2}$ has a crucial effect on the circuit noise. Therefore, the selection of low-voltage noise op-amps can effectively reduce the noise at the input. In PCB design, by avoiding long parallel traces and ensuring proper grounding, the generation of large $C_{p2}$ at the op-amp input can be suppressed. As discussed in [18], $C_{p2}$ mainly introduces a pole into the charge amplifier, which limits the operating frequency of the C-V circuit. The circuit’s Bode plot is shown in Figure 10.

**Figure 11:**
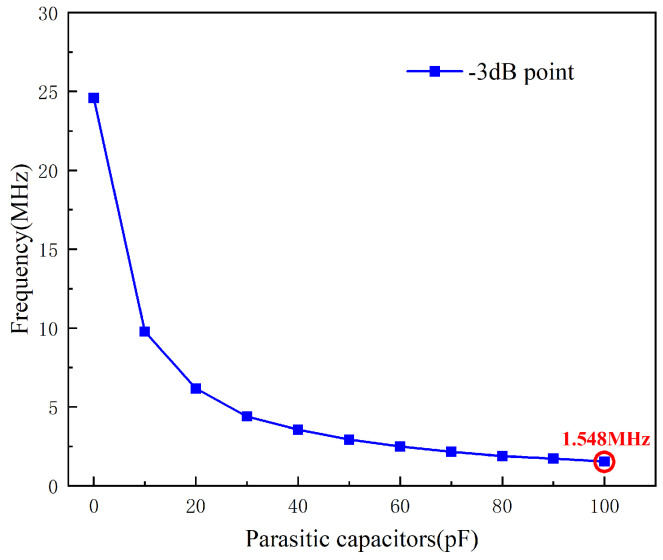
The −3 dB point affected by parasitic capacitance.

In the figure, $\omega_{u}$ represents the op-amp’s unity-gain bandwidth product. It is evident in Figure 10 that $C_{p2}$ limits the operating frequency range of the circuit. A parameter sweep was performed with $C_{p2}$ values ranging from 0 to 100 pF, and the attenuation trend of the corresponding −3 dB point is shown in the Figure 11.

It can be observed that when Cp2 = 100 pF, the −3 dB point occurs at f=1.548 MHz, which does not cause attenuation at the circuit’s operating frequency of 1 MHz. In this circuit, Cp2 is typically around 10 pF [19], which demonstrates that the circuit is immune to the effects of Cp2 on its sensitivity.

$C_{f}$ plays a crucial role in determining the gain of the C-V circuit and the circuit gain of the capacitive readout circuit [20], as shown in Equation (12). The smaller the $C_{f}$, the higher the scale factor of the circuit, but as shown in Equation (15), if $C_{f}$ is too small, it results in deterioration of the circuit’s noise performance of the circuit. A compromise to consider is that $C_{f}$ is slightly smaller than the equivalent static capacitance $C_{0}$, taking a value of 3 pF. Optimizing the circuit’s scaling factor can also theoretically be achieved by reducing $R_{1}$ in the subtraction circuit. However, taking into account the possibility of increasing noise interference, the actual circuit uses $R_{1}=R_{3}$.

In addition, considering that poor grounding increases additional noise, making the circuit more susceptible to common-mode disturbances and reducing its signal-to-noise ratio, the circuit uses the output high-precision common-mode bias voltage $V_{ref}$ from the LDO regulator as a virtual ground to shield the circuit from disturbances caused by poor grounding.

## 5. Test Results and Analysis

**Figure 12:**
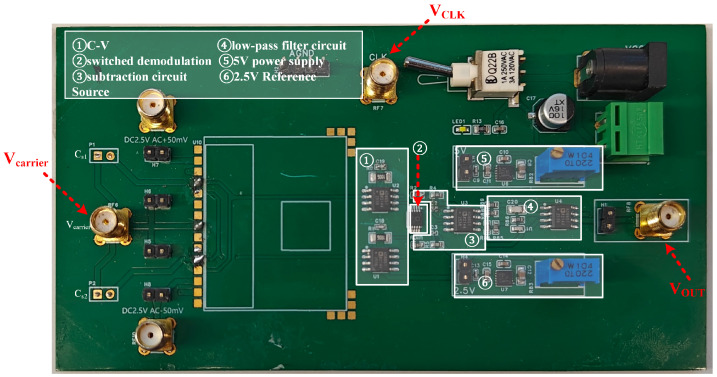
Physical diagram of the circuit.

**Figure 13:**
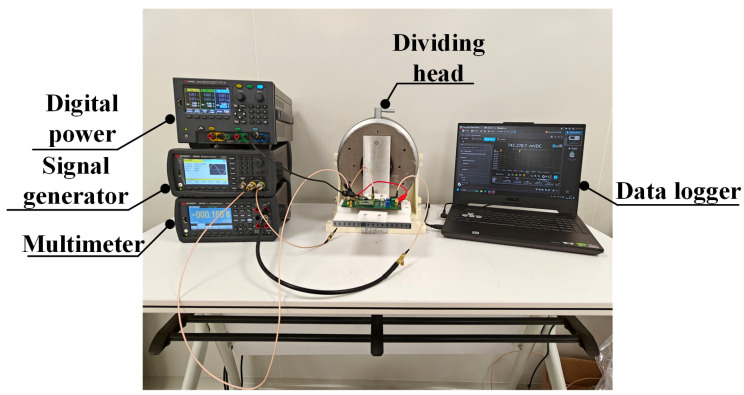
Accelerometer system test environment.

The capacitive readout circuit is depicted in Figure 12. In the figure, the carrier wave and square wave are connected via wiring terminals, and the output voltage $V_{OUT}$ is delivered through a wiring terminal to a digital multimeter for reading. The accelerometer test system was built to test and verify the designed MEMS accelerometer capacitive readout circuit, as shown in Figure 13. The dividing head shown in the figure can be precisely rotated to any angle, providing accurate angular variations for the scale factor test in the experiment. During the test, the sensitive axis of the accelerometer needs to be aligned with the rotation direction of the dividing head.

The scale factor, zero-bias stability, and noise performance indicators of the readout circuit were tested using a commercial standard MEMS accelerometer. Notably, unlike the ideal case, the frequency and amplitude of the carrier signal and the square-wave signal in the demodulation circuit exhibited temporal variations, which are unacceptable for high-precision detection applications. Typically, the frequency stability of these signals must be maintained within 1 ppm and the amplitude stability within 10 ppm [20,21]. To address this issue, the present study employed a Keysight high-precision digital waveform generator (Keysight Technologies, located in Santa Rosa, CA, USA) to produce both high-precision carrier and square-wave signals, ensuring that over a period of one year, the frequency stability is maintained within 0.1 ppm and the amplitude stability within 1 ppm, thereby guaranteeing the long-term stability of the signals.

### 5.1. Scale Factor Test

The scaling factor refers to the output voltage variation in the accelerometer when transitioning from 0 g to 1 g, representing the gain capability of the capacitive readout circuit. The circuit was tested using the four-point rolling method, where the four points correspond to angles of $0^{\circ }$, $90^{\circ }$, $180^{\circ }$, and $270^{\circ }$, which correspond to the accelerometer being at ±1 g and 0 g, respectively. The output voltages of the circuit were measured at these points, and a total of six sets of data were collected, as shown in Table 1.

The scale factor is obtained as follows:

(16) $$S_{AV}=\frac{E_{90}-E_{270}}{2g}$$

**Figure 14:**
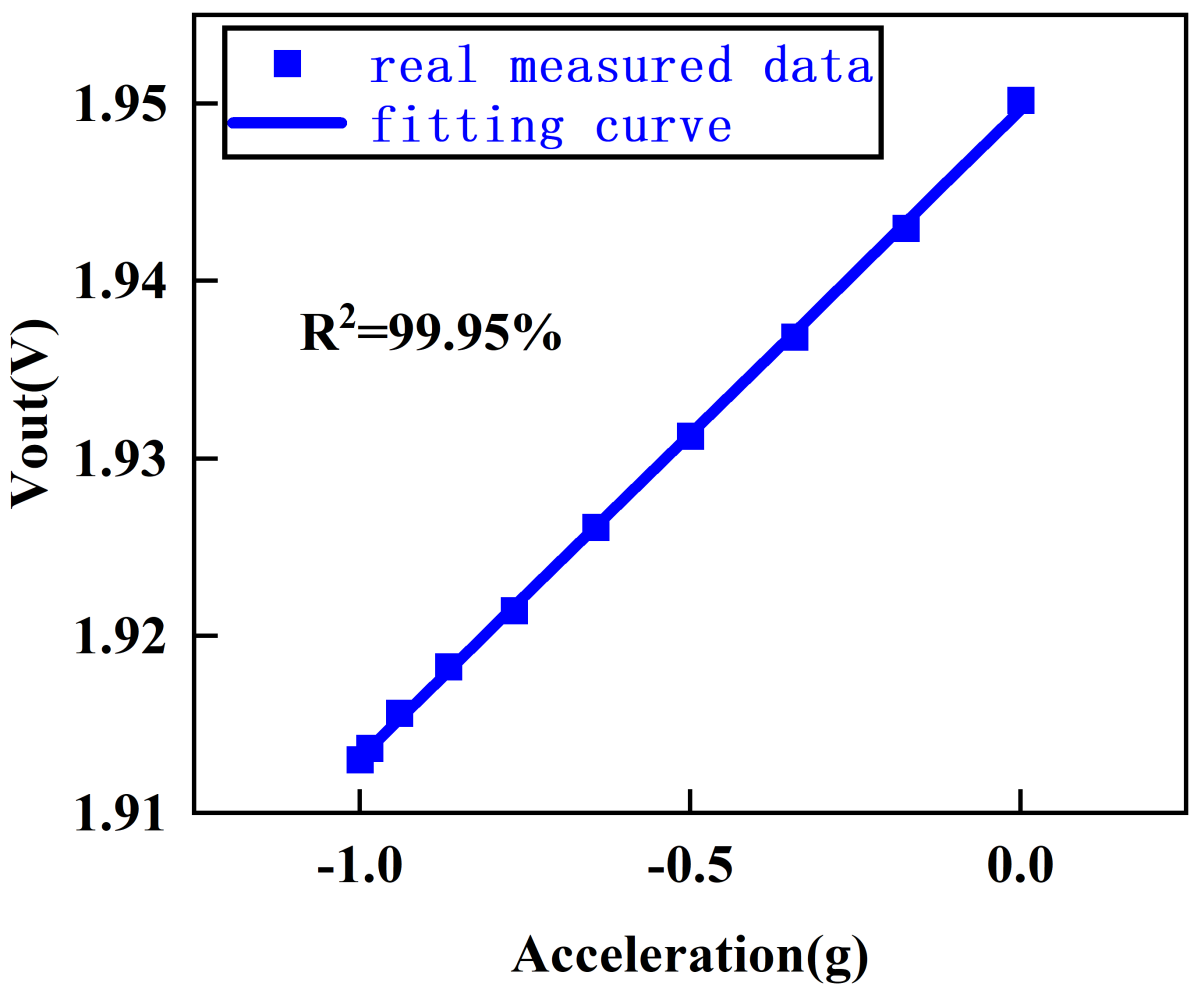
Linearity test fitting curve.

In Equation (16), $E_{90}$ and $E_{270}$ correspond to the output voltages when the accelerometer is flipped to $90^{\circ }$ and $270^{\circ }$, respectively. To prevent the circuit from exhibiting different gains for ±1 g acceleration, the output voltages for ±1 g are averaged. Additionally, the output voltages are recorded for every 10° change as the accelerometer is moved from −1 g to 0 g, and a linear fit is performed. The system’s linearity is found to be 99.95%, demonstrating that the system has good linearity (Figure 14).

### 5.2. Zero-Bias Stability Test

**Figure 15:**
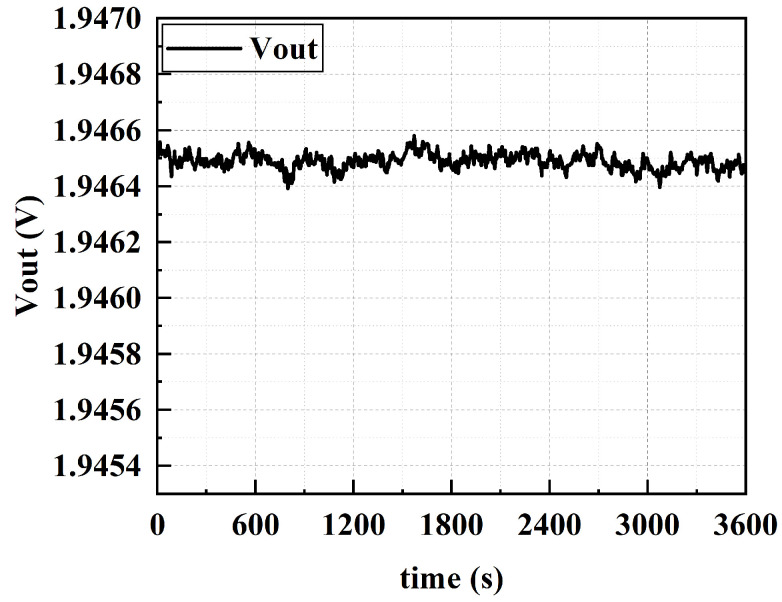
Zero-bias stability test results.

The data acquisition is set to collect the system’s output voltage value every 1 s. Figure 15 shows the test data of the accelerometer in the 0 g state. Taking the test data from 1 h after stabilization, the standard deviation of the output is 13.4 uV, and the stability of the system’s zero bias over 1 h is 0.38 mg.

### 5.3. Noise Performance Test

**Figure 16:**
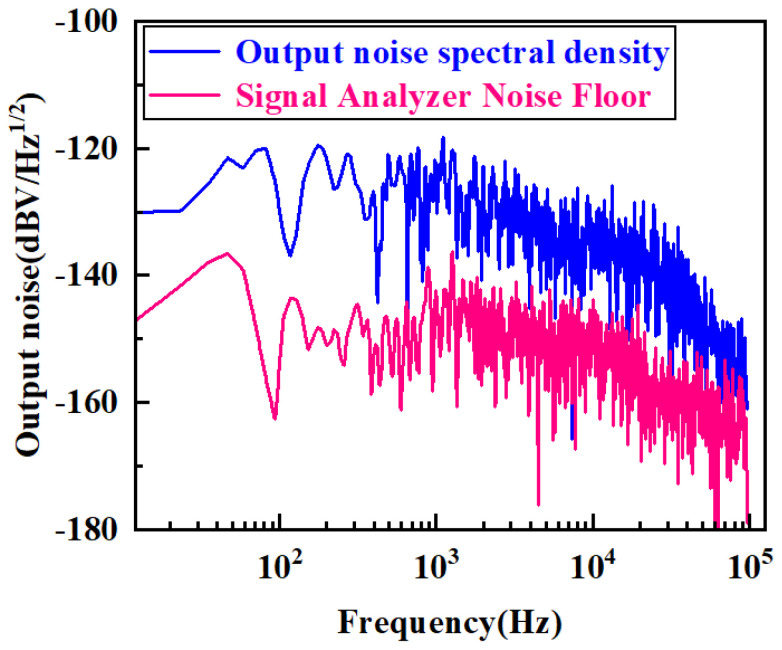
Circuit output noise power spectral density waveforms.

To test the noise performance of the circuit, an APX555 spectrum analyzer (Audio Precision, Beaverton, OR, USA) was used to measure the noise power spectral density (PSD) of the final output signal in the circuit. The test results are shown in Figure 16, in which the blue line is the PSD of the readout circuit output, and the red line is the PSD of the signal analyzer’s noise floor. As can be seen in Figure 16, the noise floor of the output of the readout circuit is $-120\;\mathrm{dBV}/{\mathrm{Hz}}^{1/2}$ ($10^{-6}\;V/{\mathrm{Hz}}^{1/2}$), and the noise floor of the signal analyzer is $-135\;\mathrm{dBV}/{\mathrm{Hz}}^{1/2}$ ($10^{-13.5/2}\;V/{\mathrm{Hz}}^{1/2}$). The circuit output noise is calculated to be $25.6\;\mu g/{\mathrm{Hz}}^{1/2}$, and the capacitive resolution is $0.103\;\mathrm{aF}/{\mathrm{Hz}}^{1/2}$, using Equations (17) and (18), respectively. Equation (19) represents the conversion relationship among $S_{AV}$, $S_{AC}$, and $S_{CV}$, where $S_{CV}$ represents the circuit’s sensitivity to capacitance.

(17) $$Noise\;floor=\frac{F_{0}-F_{SA}}{S_{AV}}$$

(18) $$Capacitive\;resolution=\frac{F_{0}-F_{SA}}{S_{CV}}$$

(19) $$S_{CV}\;=\frac{S_{AV}}{S_{AC}}$$

where $F_{0}$ is the circuit output noise floor with a value of $10^{-6}\;V/{\mathrm{Hz}}^{1/2}$, $F_{SA}$ is the signal analyzer noise floor with a value of $10^{-13.5/2}\;V/{\mathrm{Hz}}^{1/2}$, $S_{CV}$ is the circuit’s sensitivity with a value of 7.97 mV/fF, and $S_{AV}$ is the scale factor with value of 35.1 mV/g.

The capacitance readout circuit designed in this paper was compared with the circuits described in the literature, as shown in Table 2, which shows that the capacitance readout circuit designed in this paper achieves high-resolution capacitance detection, and the linearity of the system can reach 99.95%. The test results show that the circuit can meet the requirements of capacitance readout of high-precision MEMS accelerometers. Notably, the circuit is implemented on a PCB, and since most discrete components operate at supply voltages of around 5 V, the circuit’s supply voltage is inevitably higher than that of other ASIC designs.

## 6. Conclusions

This paper presents the design and implementation of an ultra-high-resolution capacitive readout circuit that employs a differential charge amplifier structure to realize the C-V circuit. Based on an analysis of the circuit’s noise spectral characteristics, this paper adopts the frequency-domain modulation technique to suppress the circuit’s low-frequency noise and common-mode noise through a differential subtractive circuit. The circuit is tested, and the test results show that at a clock frequency of 1 MHz, the capacitance resolution can reach $0.103\;{\mathrm{aF}/\mathrm{Hz}}^{1/2}$. The test results indicate that the circuit designed in this paper exhibits high-resolution capacitance and excellent noise suppression performance. However, regarding power consumption, the PCB-level implementation of this circuit exhibits relatively high power usage compared to ASIC designs. Future work will focus on redesigning the circuit as an application-specific integrated circuit (ASIC) to enhance performance while reducing power consumption, thereby better satisfying the stringent requirements for detecting minute capacitance variations in MEMS accelerometers under high-precision, low-noise conditions.

## Figures and Tables

**Table 1:** Scale factor test data.

Angle/(°)	Vout/V					
0	1.94941	1.94949	1.94900	1.94857	1.95024	1.94996
90	1.98507	1.91417	1.91343	1.91355	1.91358	1.91373
180	1.94956	1.95039	1.94804	1.94771	1.94761	1.94637
270	1.91433	1.98486	1.98343	1.98365	1.98372	1.98381

**Table 2:** Readout circuit performance comparison.

	[22]	[23]	[24]	[19]	This Work
Sensor sens. $S_{\mathrm{AC}}$ (fF/g)	15	-	-	4.5	4.4
Circuit sens. $S_{\mathrm{CV}}$ (mV/fF)	-	-	27.5	-	7.97
Capacitive resolution (aF/H$z^{1/2}$)	-	-	0.3	0.5	0.103
Noise floor (μg/H$z^{1/2}$)	290	130	81	112	25.6
Scale factor $S_{\mathrm{AV}}$ (mV/g)	-	23.3	-	-	35.1
Zero bias stability (mg)	20	-	-	-	0.38
Supply (V)	1	-	1.8	1.8	5
Bandwidth (Hz)	50	-	10,000	12,500	10,000

## Data Availability

The raw data supporting the conclusions of this article will be made available by the authors upon request.
